# Refining Diagnosis of *Schistosoma haematobium* Infections: Antigen and Antibody Detection in Urine

**DOI:** 10.3389/fimmu.2018.02635

**Published:** 2018-11-14

**Authors:** Claudia J. de Dood, Pytsje T. Hoekstra, Julius Mngara, Samuel E. Kalluvya, Govert J. van Dam, Jennifer A. Downs, Paul L. A. M. Corstjens

**Affiliations:** ^1^Department of Cell and Chemical Biology, Leiden University Medical Center, Leiden, Netherlands; ^2^Department of Parasitology, Leiden University Medical Center, Leiden, Netherlands; ^3^National Institute for Medical Research, Mwanza, Tanzania; ^4^Department of Medicine, Catholic University of Health and Allied Sciences, Mwanza, Tanzania; ^5^Center for Global Health, Weill Cornell Medicine, New York, NY, United States; ^6^Department of Medicine, Bugando Medical Centre, Mwanza, Tanzania

**Keywords:** *Schistosoma haematobium*, CAA anodic antigen, CCA cathodic antigen, antibody, urine, lateral flow assay, UCP upconverting reporter particle

## Abstract

**Background:** Traditional microscopic examination of urine or stool for schistosome eggs lacks sensitivity compared to measurement of schistosome worm-derived circulating antigens in serum or urine. The ease and non-invasiveness of urine collection makes urine an ideal sample for schistosome antigen detection. In this study several user-friendly, lateral-flow (LF) based urine assays were evaluated against a composite reference that defined infection as detection of either eggs in urine or anodic antigen in serum.

**Method:** In a Tanzanian population with a *S. haematobium* prevalence of 40–50% (*S. mansoni* prevalence <2%), clinical samples from 44 women aged 18 to 35 years were analyzed for *Schistosoma* infection. Urine and stool samples were examined microscopically for eggs, and serum samples were analyzed for the presence of the anodic antigen. Urines were further subjected to a set of LF assays detecting (circulating) anodic (CAA) and cathodic antigen (CCA) as well as antibodies against soluble egg antigens (SEA) and crude cercarial antigen preparation (SCAP).

**Results:** The urine LF anodic antigen assay utilizing luminescent upconverting reporter particles (UCP) confirmed its increased sensitivity when performed with larger sample volume. Qualitatively, the anodic antigen assay performed on 250 μL urine matched the performance of the standard anodic antigen assay performed on 20 μL serum. However, the ratio of anodic antigen levels in urine vs. serum of individual patients varied with absolute levels always higher in serum. The 10 μL urine UCP-LF cathodic antigen assay correlated with the commercially available urine POC-CCA (40 μL) test, while conferring better sensitivity with a quantitative result. Urinary antibodies against SEA and SCAP overlap and correlate with the presence of urinary egg and serum anodic antigen levels.

**Conclusions:** The UCP-LF anodic antigen assay using 250 μL of urine is an expedient user-friendly assay and a suitable non-invasive alternative to serum-based antigen testing and urinary egg detection. Individual biological differences in the clearance process of the circulating antigens are thought to explain the observed high variation in the type and level of antigen (anodic or cathodic) measured in urine or serum. Simultaneous detection of anodic and cathodic antigen may be considered to further increase accuracy.

## Introduction

Schistosomiasis is a neglected parasitic infection that may affect over 700 million people worldwide (1), and optimal diagnostic strategies have still not been established. Accurate diagnosis of an active *Schistosoma* infection requires elaborate parasitology on multiple samples as eggs cluster together and shedding is irregular (2); these factors are known to limit the sensitivity of the egg detection methods in urine (i.e., *S. haematobium*) as well as stool (i.e., *S. mansoni*). This traditional diagnostic method of microscopic examination of urine (3) or stool (4) for schistosome eggs in medium to low endemic areas lacks sensitivity compared to measurement of schistosome worm antigen in serum or urine (5, 6). Maximizing sensitivity becomes particularly pressing in the setting of low-intensity infections (more common among adults), and also as efforts to interrupt transmission accelerate (7). Schistosome circulating anodic and cathodic antigens (CAA and CCA, respectively) are glycosaminoglycan-like carbohydrates regurgitated from the gut of live schistosome worms into the host bloodstream (8). Both antigens are cleared from the human circulation via the kidneys and can be detected in serum as well as urine, allowing non-invasive diagnosis. Antigen levels diminish rapidly following anti-schistosome praziquantel treatment (9, 10). Detection of these antigens has been shown to be more accurate to identify active and low grade infections and does not require the analyses of different sample types in case of unknown species or mixed infection. A urine cathodic antigen assay is available as a low cost point-of-care test (POC-CCA) with excellent diagnostic capability for moderate to high-grade *S. mansoni* infection (5, 11, 12), but to a lesser extent for *S. haematobium* detection (13). This assay is mostly used for mapping and monitoring of endemic areas (14–16), but also showed its use in non-endemic countries for individual diagnosis of e.g. immigrants (17–19).

A more sensitive genus-specific anodic antigen LF assay, applicable for all *Schistosoma* species including veterinary infections, was developed several years ago (20). Multiple studies have utilized this up-converting phosphor LF assay (UCP-LF CAA) to investigate prevalence in *S. mansoni, S. haematobium, S. mekongi*, and *S. japonicum* settings using serum, urine, or both (21–29). The test platform includes a sample preparation step that allows analysis of increased sample volume providing ultimate sensitivity down to detection of single worm (single sex) infections. A study in the People's Republic of China showed a sensitivity of >90% for diagnosis of *S. japonicum* using the assay on concentrated urine samples, as compared to a composite reference that defined infection as detection of either eggs or anodic antigen in serum or urine (30). Anodic antigen concentrations were approximately 10-fold lower in urine than in serum similar to what has been described previously (31).

We sought to determine the performance of several user-friendly LF based urine assays in a population in which *S. haematobium* is endemic. Urine LF assays were evaluated against a composite reference of either detecting eggs in urine and/or schistosome anodic antigen in serum. Eggs were detected by standard urine filtration microscopy and the level of schistosome anodic antigen in serum was determined using the UCP-LF CAA assay. We also compared these findings to the commercially-available POC-CCA urine test, as well as an experimental UCP-LF urine assay, both detecting the cathodic antigen. In addition, two rapid UCP-LF antibody assays detecting antibodies against crude cercarial and soluble egg preparations were evaluated for use with urine samples.

## Methods

### Study site and population

This study was conducted in four rural inland villages in northwest Tanzania in which *S. haematobium* is highly endemic (32). The prevalence of *S. haematobium* infection among adults in these villages is ~40–50%, and the prevalence of *S. mansoni* infection is ≤2% (25). All women between the ages of 18 and 35 in each village were invited to receive free screening for schistosomiasis. Women provided urine, stool, and blood samples. Urine and stool were examined microscopically for *Schistosoma* eggs, and serum was used to determine schistosome anodic antigen concentration. Urine samples were further analyzed for anodic and cathodic antigens and anti-schistosome antibodies. Women were also screened for HIV and those enrolled in this study were all HIV-uninfected. Individuals found to be HIV-infected were given referral letters to the nearest HIV Care and Treatment Centre, where they could access HIV care free of charge.

### Sample collection and field analysis

All samples were collected between 10:00 a.m. and 2:00 p.m. on study enrollment days. Women provided at least 25 mL of urine. Ten mL of urine was filtered in the field and immediately examined microscopically for *S. haematobium* eggs. Fifteen mL of urine was placed into Falcon tubes and stored at −20°C at the reference laboratory in Mwanza until transport to Leiden University Medical Center for schistosome antigen and antibody testing. To test for *S. mansoni* eggs, five Kato-Katz slides were prepared from each stool sample, a strategy with a sensitivity comparable to testing three stool samples obtained on separate days (33). Serum was centrifuged and separated each day upon return to Mwanza and subsequently stored at −20°C.

### Serum anodic antigen testing in local laboratory

Serum anodic antigen testing using the (UCP-LF) technology was performed at the National Institute for Medical Research in Mwanza as previously described (20, 34). In this dry-reagent format of the assay, 20 μL of serum was used (SCAA20) (12). Serum was pretreated with an equal volume of 4% (v/v) trichloroacetic acid (TCA); applied cut-off thresholds are indicated in Table 1. Cut-offs are derived from Corstjens et al. (12) and are determined from testing series of negative samples.

**Table 1:** Cut-off thresholds applied for the UCP-LF antigen assays.

	**Cut-off threshold**		
	**Positive (pg/mL)**	**Indecisive (pg/mL)**	**Negative (pg/mL)**
Anodic antigen test*^a^*			
SCAA20	>30	10–30	<10
UCAA10	>30	10–30	<10
UCAA250	>3	1–3	<1
UCAA2000	>0.3	0.1–0.3	<0.1
Cathodic antigen test*^b^*			
UCCA10	>2,460		<2,460

a *Anodic antigen assays utilizing the UCP-LF test platform: SCAA, CAA assay performed on 20 μL serum; UCAA, CAA assay preformed on respectively 10, 250 and 2000 mL urine*.

b *Cathodic antigen assay utilizing the UCP-LF test platform: UCAA, CCA assay performed on 10 μL urine*.

### Urine anodic antigen lateral flow assays

UCP-LF anodic antigen assays using increasing volumes of urine were performed at Leiden University Medical Center as previously described (35). All urine samples were tested using 10 μL of urine (UCAA10) and 250 μL of urine (UCAA250) (12). Samples with negative or indecisive results in the UCAA250 assay were retested using 2,000 μL of urine (UCAA2000). All samples were extracted with an equal volume of 4% (v/v) TCA. Concentration of the clear supernatant was performed using 0.5 and 4 mL single-use 10 kD concentration devices (Amicon Ultra Centrifugal Filters, Millipore Corp) for the UCAA250 and UCAA2000, respectively. The resulting 20 μL concentrate was used in the assay. Applied cut-off thresholds are indicated in Table 1.

### Additional urine tests

Besides the UCP-LF anodic antigen assay, urine samples were also examined with four additional tests. The commercially-available urine point-of-care (POC)-CCA test (Rapid Medical Diagnostics, Pretoria, South Africa; format 2013, utilizing chase buffer) was used to evaluate its performance in an *S. haematobium*-endemic setting. Furthermore, a prototype UCP-LF cathodic antigen assay, quantitating cathodic antigen in urine, was performed with 10 μL urine (UCCA10), following the same protocol as described for the anodic antigen assay (UCAA10), but with a cathodic antigen specific UCP reporter conjugate and LF strips, as described earlier (12). Cut-off thresholds for the UCCA10 assay are indicated in Table 1. The UCP-LF cathodic antigen assay has not been explored with concentrated urines and therefore does not include an indecisive (potential positive) range; the applied cutoff is according to the threshold as determined previously with ELISA (36). The UCCA10 utilizes the same antibody on the UCP reporter as on the LF strip (mouse monoclonal 54-4C2), whereas the POC-CCA uses a second antibody on the LF strip (mouse monoclonal 54-5C10). Sample input differs as the POC-CCA requires 40 μL of untreated urine whereas the UCCA10 assay utilizes 10 μL urine which is extracted with an equal volume of TCA (4% w/v). In addition, two UCP-LF-based assays for antibody detection against soluble egg antigens (SEA) and crude cercarial antigen preparation (SCAP) in urine were applied, as previously described (12). The cut-off threshold was calculated from 11 samples that were negative in all antigen assays as well as with microscopy.

### Data analysis

Sensitivity (Sn) and specificity (Sp) were calculated for each diagnostic test, considering the combined results from microscopy (egg count) and SCAA20 (serum anodic antigen) as the “composite reference.” Hence, any positive test result by urinary microscopy or SCAA20 (indecisive results considered as negative) was considered a true-positive result. Consequently, the specificity was set to be 100% for these two assays. To indicate the weight of indecisive (potentially positive) and trace results, Sn and Sp were calculated for each test separately including indecisive or trace results either as positive or negative. Additionally, the performance of each test was compared to UCAA2000 and a combination of UCAA2000 and UCCA10 (indecisive results of the UCAA2000 were considered as negative). The correlation between the two antibody assays was calculated using the Spearman rho. All statistical analysis were performed using IBM SPSS Statistics v 23.0 (IBM Corp, Armonk, NY).

### Ethics

This project was approved by the Catholic University for Health and Allied Sciences/Bugando Medical Centre (Mwanza, Tanzania), the National Institute for Medical Research (Dar es Salaam, Tanzania), and Weill Cornell Medical College (New York, New York). All study participants provided written informed consent and were given praziquantel on the day of study enrollment in accordance with World Health Organization recommendations for treatment of adults in areas in which schistosome infections are highly endemic (37).

## Results

### Serum anodic antigen and urine microscopy findings—A composite reference

Between June and August 2013, we obtained 39 samples from women who presented for schistosomiasis screening, and 5 additional post-treatment samples from women with a confirmed *S. haematobium* infection and who received praziquantel treatment 2 months previously. The distribution of locally determined serum anodic antigen levels (SCAA20) and urine microscopy egg-count results are shown in Table 2. Of the 44 samples, 14 were egg and serum anodic antigen positive. The serum anodic antigen assay detected 3 additional positives with concentrations ≥30 pg/mL; 8 other samples were ranked indecisive of which one had 2 eggs per 10 mL. In total, 18 women met the criteria for the composite reference of *S. haematobium* infection, defined as either a positive test result by urine egg microscopy or a serum anodic antigen level >30 pg/mL. *S. mansoni* eggs in stool or urine were not identified in any study participant.

**Table 2:** Urine egg microscopy and serum CAA results for 44 individuals.

		**Urine egg microscopy**		**Total**
		**Positive (*n* = 15)**	**Negative (*n* = 29)**	**(*n* = 44)**
SCAA20	Positive	14^*^	3^*^	17
	Indecisive	1^*^	7	8
	Negative	0	19	19

* *Numbers indicate samples included in the composite reference: urine egg microscopy positive and/or a SCAA20 result >30 pg/mL*.

### Urine anodic antigen (UCAA) assay results—performance utilizing large sample volume

**Figure 1:**
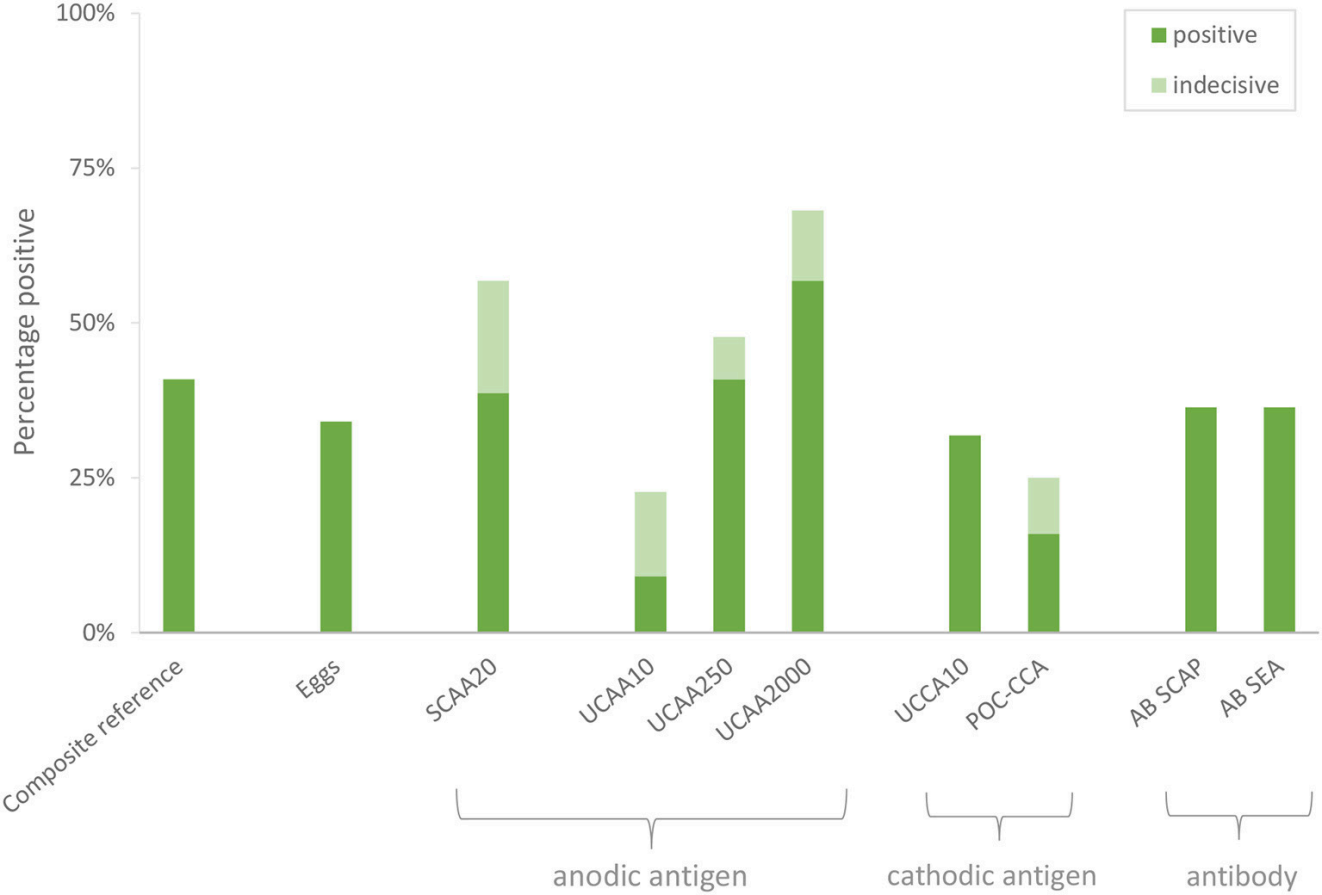
Performance of individual diagnostic tests to identify *S. haematobium* infection. A composite reference was defined, based on assays performed locally, ranking samples positive based on either a positive test result by urine egg microscopy and/or a serum anodic antigen level >30 pg/mL.

Urine samples transported to LUMC (the Netherlands) were analyzed utilizing increased sample volume for enhanced sensitivity as well as to resolve the status of samples with indecisive results. Applying the dry reagent cutoff threshold of 30 pg/mL (12), the UCAA10 assay was positive for 4 out of the 44 samples, and indecisive for 6. These 10 positive/indecisive results were all part of the 18 composite reference positives (Table 3). The 25-fold larger volume UCAA250 assay identified 16 out of 18 composite reference positives, 2 additional positives not detected by the UCAA10 assay, and another 3 as indecisive. The 200-fold-larger volume UCAA2000 assay correctly identified all 18 composite reference positives, as well as 7 additional positives that were negative by both urine microscopy and serum anodic antigen. Additionally, 5 indecisive samples were obtained with the UCAA2000, one also scoring a SCAA20 indecisive result (13 pg/mL). All samples positive with a lower volume UCAA assay remained positive when retested at a higher volume. Results are summarized in Figure 1 showing the percentage of positive and indecisive samples for all applied diagnostics.

**Table 3:** Performance of the increased volume UCP-LF anodic antigen assays vs. the composite reference.

		Composite reference^*^		**Total**
		**Positive (*n* = 18)**	**Negative (*n* = 26)**	**(*n* = 44)**
UCAA10	Positive	4	0	4
	Indecisive	6	0	6
	Negative	8	26	34
UCAA250	Positive	16	2	18
	Indecisive	0	3	3
	Negative	2	21	23
UCAA2000	Positive	18	7	25
	Indecisive	0	5	5
	Negative	0	14	14

* *Composite reference: urine egg microscopy positive and/or a SCAA20 result >30 pg/mL*.

### Urine cathodic antigen results

In this *S. haematobium* endemic setting the POC-CCA test identified 7 positives and 4 traces. These results are in agreement with the results of the prototype UCP-LF cathodic antigen assay (UCCA10). All 7 POC-CCA positives and the 3 out of 4 traces tested positive with the UCCA10 assay, which overall indicated 14 positive samples (Table 4). One UCCA10 negative sample with a POC-CCA trace result tested positive for anodic antigen in serum as well as in urine. The POC-CCA identified only 4 out of 18 composite reference positives, and of the 4 traces, only 1 was also positive by the composite reference. Table 5 shows the cathodic antigen (UCCA10) and the anodic antigen (UCAA2000) assay results. From the 25 UCAA2000 positives, 9 were also identified with the UCCA10, while the latter identified 3 additional positives that were not identified by the UCAA2000, and which were also negative according to the composite reference. For the few samples with a quantified level of both antigens, there was no obvious correlation observed between the absolute levels of the anodic and cathodic antigen in urine.

**Table 4:** Performance of the cathodic antigen assays: POC-CCA vs. the UCCA10 test.

		**UCCA10**		**Total**
		**positive (*n* = 14)**	**negative (*n* = 30)**	**(*n* = 44)**
POC-CCA	Positive	7	0	7
	Trace	3	1	4
	Negative	4	29	33

**Table 5:** Performance of the UCCA10 vs. the UCAA2000 test.

		**UCCA10**		**Total**
		**Positive (*n* = 14)**	**Negative (*n* = 30)**	**(*n* = 44)**
UCAA2000	Positive	9	16	25
	Indecisive	2	3	5
	Negative	3	11	14

### Performance of the urine anodic and cathodic antigen tests

Table 6 shows the clinical performance of each test compared to the composite reference. By definition, the clinical specificity (Sp) of both microscopy and SCAA20 is 100%. Clinical sensitivity (Sn) of anodic antigen detection in urine increases when using larger sample volume. Sp decreases with the increased urine volume as a consequence of detecting antigen positives that were not identified with the assays used to define the local composite reference. Sn and Sp values when using the assumed most sensitive urine antigen diagnostics as a gold standard are indicated in Table 7. Values are calculated for either the anodic antigen only, or for the combined anodic and cathodic antigen detection results. To indicate the weight of indecisive and trace results, Sn and Sp were calculated either including indecisive or trace results as positive or negative. Table 7 indicates Sn and Sp against the UCAA2000 and the UCAA2000 combined with the UCCA10. In this table indecisive results obtained with the UCAA2000 are categorized as negative as larger volume testing was not applied to resolve their status.

**Table 6:** Sensitivity and specificity of individual diagnostic tests vs. the composite reference.

	**With indecisives considered to be negative**		**With indecisives considered to be positive**	
	**Sn**	**Sp**	**Sn**	**Sp**
Microscopy*^a^*	83%	100%	n/a	n/a
SCAA20*^a^*	94%	100%	n/a	n/a
UCAA10	22%	100%	56%	100%
UCAA250	89%	92%	89%	81%
UCAA2000	100%	73%	100%	54%
UCCA10	33%	69%	n/a	n/a
POC-CCA	22%	88%	33%	81%

a Sp is 100% by definition as the composite reference assumes 100% specificity for microscopy and SCAA20 (in total 18 positives)

**Table 7:** Sensitivity and specificity vs. UCAA2000 and vs. UCAA2000 + UCCA10 combined.

	**UCAA2000*^a^***				**UCAA2000**+**UCCA10*^b^***			
	**Indecisives considered negative**		**Indecisives considered positive**		**Indecisives considered negative**		**Indecisives considered positive**	
	**Sn**	**Sp**	**Sn**	**Sp**	**Sn**	**Sp**	**Sn**	**Sp**
Microscopy	60%	100%	n/a	n/a	50%	100%	n/a	n/a
SCAA20	68%	100%	96%	95%	57%	100%	83%	100%
UCAA10	16%	100%	40%	100%	13%	100%	33%	100%
UCAA250	72%	100%	80%	95%	60%	100%	67%	93%
UCAA2000	100%	100%	80%	95%	83%	100%	90%	79%
UCCA10	36%	74%	n/a	n/a	47%	100%	n/a	n/a
POC-CCA*^c^*	20%	89%	32%	84%	23%	100%	37%	100%

a Assuming 100% specificity of the anodic antigen detection by the UCAA2000 (total of 25 positives when indecisive are considered to be negative)

b Assuming 100% specificity of the anodic antigen detection by the UCAA2000 and cathodic antigen detection (UCCA10) (total of 30 positives when indecisive are considered to be negative)

c POC-CCA trace results fall under indecisive

### Serology status determined through antibody detection in urine

**Figure 2:**
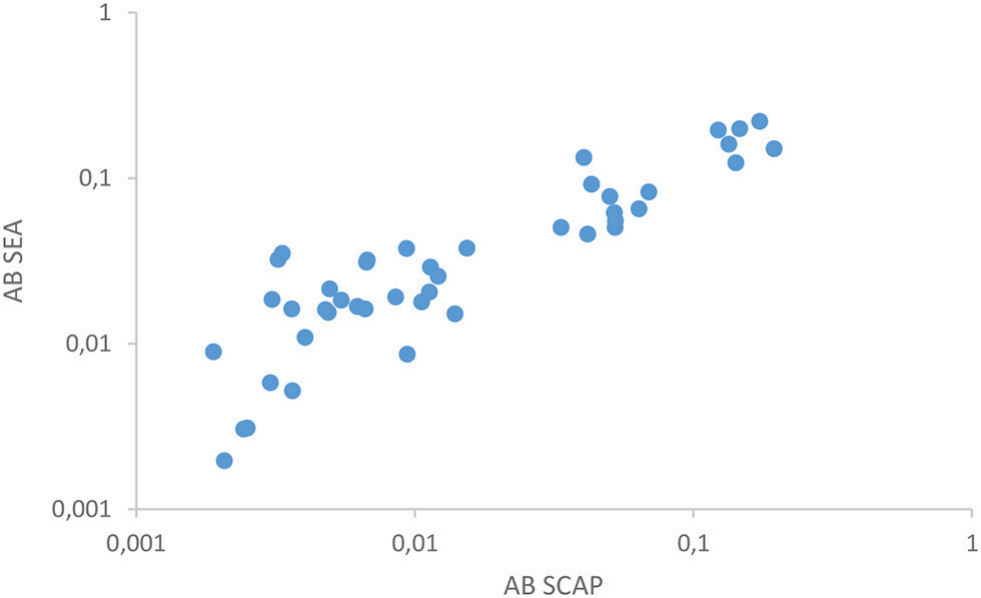
Correlation between SEA and SCAP antibodies.

The two antibody detection assays in urine correlated well (Figure 2) as was similarly demonstrated in serum in a previous study (12). Out of the 18 composite reference positives 14 showed clear reactivity with at least one of the antibody assays. Serology turned out to be negative in two egg positive cases. One of these samples (7 eggs per 10 mL urine) had a remarkably high level of the anodic antigen in serum and cathodic antigen in urine, respectively >10,000 and 5,900 pg/mL as determined with the SCAA20 (serum) and UCCA10 (urine) assays. In contrast, the urine anodic antigen level was 27.8 pg/mL (UCAA250), more than 100-fold less than the concentration in serum. This sample was also easily identified with the POC-CCA test. The second sample (5 eggs per 10 mL urine) was negative for urine cathodic antigen, but positive for anodic antigen with levels in serum and urine showing a 50-fold difference (50 and 1 pg/mL, respectively). Tentatively, urine antibody assays generally seem reactive at anodic antigen levels above 100 pg/mL in serum as well as with the presence of urinary eggs.

### Status of previously infected, praziquantel-treated participants

The serum anodic antigen assay (SCAA10) indicated that 3 of the 5 previously infected and treated individuals were still carrying an active infection, with levels ranging between 10 and 100 pg/mL. This was confirmed by urine anodic antigen, indicating levels between 1 and 15 pg/mL. A fourth sample was only positive for urine cathodic antigen, with a level of 4,500 pg/mL, while the POC-CCA test was negative. Eggs were not detected in any of the 5 individuals, but based on the outcome of these tests, 3 individuals remained positive by the composite reference and the individual with the positive UCCA10 is likely still infected as well. Thus, 4 of the 5 recently-treated women would require an additional drug treatment.

## Discussion

Although the number of individuals included in this study was relatively small (a study limitation), it clearly demonstrates that the use of larger volumes of urine enhances the sensitivity of diagnosis with the anodic antigen assay in individuals with *S. haematobium* infection. Diagnostic tests performed in Tanzania identified 18 who met the criteria for the composite reference, defined here as being urine egg microscopy positive and/or serum anodic antigen (SCAA20) positive. As a non-invasive and thus patient-friendly alternative, the UCAA250 assay utilizing 250 μL could be considered. This assay identified 18 positives, 16 of whom were identified by the composite reference. Allowing for a less than optimal specificity, indecisive results could be interpreted as positive, which would yield an additional 3 positive scores. The higher volume format UCAA2000 confirmed that this would be correct for 2 out of the 3 indecisive samples. The UCAA250 assay would then have successfully detected 20 positives out of the 25 samples that tested positive with the UCAA2000 anodic antigen assay. The UCAA2000 assay identified all 18 composite reference positive samples as well as 7 additional positives. However, this assay is more time-intensive and costly as it requires larger tubes and concentration devices that are no longer compliant with micro-centrifuges and tubes. Therefore, we would recommend accepting a slightly lower sensitivity by using 250 μL urine rather than 2000 μL. Alternative lower-cost methods to concentrate the CAA from large urine volume are being explored to improve field applicability of the UCAA2000 (38).

Utilization of a single, non-invasive urine sample, rather than performing both urine microscopy and serum anodic antigen testing, is more user-friendly, less prone to error and likely more economical. Moreover, because the anodic antigen is genus-specific, collection and testing of urine only is sufficient for diagnosis of all *Schistosoma* species infections. The same may hold for the cathodic antigen if concentrated, although this antigen has some additional biological activity due to homology to Lewis X structures (39).

The commercially available POC-CCA test for urine samples is recommended for diagnosis of *Schistosoma mansoni* infections. Use of this assay for diagnosis of *S. haematobium* infections shows variable results with generally lower performances than for *S. mansoni* (13, 40–43). Our study also documented a lower sensitivity of this test for the diagnosis of urinary schistosomiasis. This might be due to lower levels of cathodic antigen produced by *S. haematobium* worms as compared to *S. mansoni* (unpublished observations). Use of the more sensitive UCP-LF cathodic antigen assay (UCCA10) indeed increased the number of positive samples to 14 compared to 7 for the POC-CCA. Importantly, of 4 samples generating a POC-CCA trace result, 3 samples tested positive with the UCCA10 assay. Clearly, weak signals (traces) in visually interpreted tests as the POC-CCA may be more difficult to interpret. However, considering traces as negative resulted in a higher specificity, but subsequently lower sensitivity, which may reduce the usefulness of the test for mapping purposes (44, 45). Overall, the overlap between POC-CCA and UCCA10 results confirm the specificity of the POC-CCA test. The UCP-LF cathodic antigen test was developed to increase analytical sensitivity through the application of a luminescent reporter, and to explore the effect of TCA treatment on the biological background. Further development of this assay into a high sensitivity format, identical to the UCP-LF anodic antigen assay and potentially utilizing larger volumes of urine, may provide better insight on the ratio of the levels of both antigens in urine samples. When the cathodic antigen assay is amenable to larger volume testing, a duplex LF strip detecting both antigens in a single sample will further increase sensitivity. Moreover, the relation between the two antigens might provide some information regarding the infecting species.

When using (rapid) LF based assays, indecisive test results remain an important issue. For visual assays, weak signals may be scored as trace by some observers whereas others would score it as negative. Whether a trace is false positive or true positive may be difficult to determine without alternative diagnostics. The intensity of the signal might also vary per production batch of test strips, as well as whether the manufacturer is targeting highest sensitivity or specificity of the test. Further, the choice to rank trace results as a positive or negative will depend on the goal of the survey (prevalence studies vs. elimination studies) as well as potential complications of drug treatment. A strength of the UCP-LF test format is that it utilizes a luminescent reporter label that requires a reader (scanner) to analyze the LF strip and generate the test result. Besides providing extra sensitivity as compared to a visual test, this reader removes operator interpretation flaws. Moreover, the reader can be set either to include or exclude indecisive results as positive. This allows manufacturers to optimize the test for sensitivity and adjust for specificity by programming the reader.

In contrast to the detection of eggs and circulating worm antigens, the presence of host antibodies against *Schistosoma* derived biomolecules is not necessarily a measure for ongoing active infection, except in those who would not previously have been exposed to *Schistosoma* parasites [i.e., individuals from non-endemic settings or travelers; (46)]. In the current small endemic cohort, antibody detection in urine could not contribute to resolving disputes regarding anodic antigen negatives and cathodic antigen positives or indecisive/trace results as the analytical sensitivity of the assay was not sufficient to identify all infected individuals. This may have been a consequence of setting relatively high cutoff thresholds. Studies with larger cohorts are needed to determine accurate values; appropriate thresholds may also provide insight in potential differences of reactivity against SCAP and SEA. Although the assay is not yet fully optimized, this study has demonstrated that the detection of specific host antibodies in non-invasive urine samples is feasible. This would constitute a rapid and low cost assay, but it remains to be determined whether urine would be a good enough alternative to the more invasive fingerstick blood.

## Conclusion

The non-invasive UCP-LF anodic antigen (CAA) assay utilizing 250 μL of urine has been demonstrated to be a valid alternative for the current locally used diagnostic strategy, which is based on egg microscopy combined with anodic antigen serum analysis. Furthermore, testing for the presence of the worm derived anodic antigen in large volume urine samples is a sensitive tool to monitor treatment efficiency. An important feature of the UCP-LF antigen urine assay is the potential confirmation of indecisive test results by using larger sample volumes. Testing for the presence of the cathodic antigen (CCA) would have added value also in *S. haematobium* settings, although more studies are needed to validate the status of anodic antigen negative and cathodic antigen positive samples. Anticipated development of a UCP-LF duplex test combining the anodic and cathodic antigen may improve sensitivity.

## Author contributions

JD and PC designed the study. CdD was responsible for the production of all UCP-LF test materials and performed UCP-LF urine assay. JD, JM, and SK were responsible for the collection of samples and Schistosoma diagnostics performed in Tanzania. CdD, PH, GvD, JD, and PC performed the data analysis and interpretation of the results. All authors were involved in drafting the manuscript and approved the final version.

### Conflict of interest statement

The authors declare that the research was conducted in the absence of any commercial or financial relationships that could be construed as a potential conflict of interest.
